# Performance analysis and modelling of circular jets aeration in an open channel using soft computing techniques

**DOI:** 10.1038/s41598-024-53407-3

**Published:** 2024-02-07

**Authors:** Diksha Puri, Raj Kumar, Sushil Kumar, M. S. Thakur, Gusztáv Fekete, Daeho Lee, Tej Singh

**Affiliations:** 1 School of Environmental Science, Shoolini University, Solan, Himachal Pradesh 173229 India; 2 Department of Mechanical Engineering, Gachon University, Seongnam, 13120 South Korea; 3 Department of Physics, Hansraj College, University of Delhi, Delhi, 110007 India; 4 Department of Civil Engineering, Shoolini University, Solan, Himachal Pradesh 173229 India; 5 Department of Material Science and Technology, Széchenyi István University, 9026 Győr, Hungary; 6 Savaria Institute of Technology, Faculty of Informatics, ELTE Eötvös Loránd University, Budapest, 1117 Hungary

**Keywords:** Engineering, Mathematics and computing

## Abstract

Dissolved oxygen (DO) is an important parameter in assessing water quality. The reduction in DO concentration is the result of eutrophication, which degrades the quality of water. Aeration is the best way to enhance the DO concentration. In the current study, the aeration efficiency (E_20_) of various numbers of circular jets in an open channel was experimentally investigated for different channel angle of inclination (θ), discharge (Q), number of jets (J_n_), Froude number (*Fr*), and hydraulic radius of each jet (HR_Jn_). The statistical results show that jets from 8 to 64 significantly provide aeration in the open channel. The aeration efficiency and input parameters are modelled into a linear relationship. Additionally, utilizing WEKA software, three soft computing models for predicting aeration efficiency were created with Artificial Neural Network (ANN), M5P, and Random Forest (RF). Performance evaluation results and box plot have shown that ANN is the outperforming model with correlation coefficient (CC) = 0.9823, mean absolute error (MAE) = 0.0098, and root mean square error (RMSE) = 0.0123 during the testing stage. In order to assess the influence of different input factors on the E_20_ of jets, a sensitivity analysis was conducted using the most effective model, i.e., ANN. The sensitivity analysis results indicate that the angle of inclination is the most influential input variable in predicting E_20_, followed by discharge and the number of jets.

## Introduction

As a result of growing human populations and climate change, aquatic environments are going through unprecedented changes. Water quality deteriorates due to climate change, damaging aquatic life^1^. Aquaculture, food production, environmental monitoring, and industrial production are just a few sectors that depend on dissolved oxygen (DO) concentration, which is an essential water quality indicator^2^. Typically, the amount of DO in water is stated in terms of milligrams per litre (mg/L). The ideal DO for high-quality water is 5–6 mg/L^3^. Water quality deteriorates, and there is mass fish mortality if the DO drop level falls below 2 mg/L^4^. Biological treatment is used in sewage and water treatment facilities via water jet oxygenation systems to effectively manage and regulate human and animal waste^5^. Numerous approaches, including physical procedures like adsorption and membrane filtration, chemical procedures like Fenton oxidation and electrochemical oxidation, and numerous biological methods, have been developed to treat wastewater^6^. Alternative options for raising water quality include hydraulic structures like stepped spillways, nozzle orifices, or free overflow structures. Weirs, venturi aerators, stepped cascades, and stepped spillways can all increase the amount of dissolved oxygen in a river flow system. Drop structures, including baffle blocks, chutes, weirs, and cascades, are frequently employed in straight-flow canals. Recently, research has been conducted to study the air/water flow ratio (Qa/Qw) and E20 in different hydraulic structures^7–9^. Many researchers have been conducted to study air entrainment by plunging water jets. Experimental studies on air entrainment by plunging water jets were carried out by researchers^10–12^. Many researchers have studied the increase in dissolved oxygen in weirs. Gameson^13^ was the first to document the river weir's potential for aeration. Since then, several laboratory studies on weir aeration have been conducted^14–19^. The research has also been conducted on Parshall and venturi flumes, improving DO concentration. A Parshall flume is a dynamic instrument because of its wide range of uses in wastewater treatment plants, mine discharge, irrigation canals, and dam seepage^20^. Many researchers conducted experiments on E_20_ at Parshall flumes^21,22^.

Several natural instances include plunging jets from rectangular weirs, sluices, and other comparable water systems that oxygenate or capture oxygen from the air to purify falling or running water.

The instant rate of change in DO concentration ($dC/dt$) is given by the Eq. (1) as follows:

1 $$\frac{dc}{dt}={K}_{L}\frac{{A}_{S}}{V}({C}_{Sat}-C),$$

where the saturation and DO concentrations, respectively, are ${C}_{Sat}$ and $C$. Oxygen's fluid film coefficient is ${K}_{L}$, where ${A}_{s}$ is surface-area and V is volume.

The water-atmospheric partition is used for the prediction relating to the ${C}_{Sat}$. In the event that the presumption is accurate, ${C}_{Sat}$ stays steady throughout the time, and E (oxygen transfer efficiency) can be calculated as follows:

2 $$E=\frac{{C}_{down}-{C}_{up}}{{C}_{sat}-{C}_{up}}.$$

The subscripts ‘up’ and ‘down’ indicate up-stream and down-stream locations of the jet screen, respectively.

The ratio of oxygen transferred to water to oxygen that could theoretically be ejected into the water in ideal conditions is known as aeration efficiency. The aeration efficiency (E_20_) is 100% or one when all the oxygen that might possibly be transported to the water is actually transferred. When no dissolved oxygen is transferred, E_20_ is zero. The following equation of the correction factor is used to preserve uniformity in measured experiments and standardise the results acquired at various temperatures to 20 °C. The adjustment factor accounts for the variations in how soluble oxygen is in water at various temperatures are presented in Eqs. (3) and (4)^23^. The experiments were performed within the water temperature (T) range of 23 °C–25 °C.

3 $$1-{E}_{20}={(1-E)}^{1/f}$$

Following is the formula for calculating the aeration exponent, $f$, and the oxygen transfer efficacy at 20 °C, E_20_:

4 $$f=1+2.1\times {10}^{-2}\times \left(T-20\right)+8.25\times {10}^{-5}\times ({T-20)}^{2}.$$

Several researchers have studied the oxygen diffusion between air and water caused by falling jets^24^. It was discovered that the impact angle had very little effect on the volumetric oxygen transfer coefficient^25^. The air/water oxygen transfer in the biological aerated filter was studied^26^. The liquid properties that affected the speeds at which oxygen and air were carried in plunging jet reactors were examined^27^. Multiple falling jets for oxygen transport were described by Deswal and Verma^28^. Chanson and Brattberg^29^ researched air entrainment via a two-dimensional plunging jet, while Deswal and Verma^30^ investigated air/water oxygen transfer in an aerated biological filter. The authors have demonstrated in the experiments^5,31^ that nozzle shapes, or jet geometry, affect air absorption and oxygen transport.

Hydraulics research has historically been conducted using experimental formulas, mathematical models, and physical tests. These tests are simple, but they take a lot of time and often yield inaccurate results. Solutions to problems faced in hydraulics engineering, such as predicting aeration efficiency, have emerged with the advent of soft computing. Soft computing models have drawn a lot of attention in engineering^32–36^ because they can use historical data to learn the complex correlations between different factors and then use that information to generate precise predictions on new data. The topic of aeration has demonstrated the usefulness of soft computing techniques. Adaptive neurofuzzy inference system (ANFIS) and least square support vector machines have been successfully applied by Baylar et al.^37^ to data sets of air-entraining rate and aeration efficiency obtained from descending overfall jet from triangular-weir. Multiple linear and multiple nonlinear regression-based predictive equations were employed to compare the efficacy of various modelling techniques. Bagatur and Onen^38^ investigated the ability of genetic expression programming (GEP)as a substitute to forecast the design coefficient in an ogee-crested spillway. Support vector machines (SVM) and GEP techniques were used by the authors to correctly forecast the volumetric oxygen transfer coefficient of numerous plunging jets descending into a still water pool^39^. To predict the volumetric oxygen transfer coefficient by vertical and angled multiple jets, GEP modelling was utilized to assess the kernel functions based on support vector and multi linear regressions^40^. Using ANN (artificial neural network) and nonlinear regression techniques, Kramer et al.^41^ successfully evaluated the penetration depth of plunging water jets with extended discharge. Kumar et al.^42^ predicted volumetric oxygen transfer coefficient with soft computing models such as ANN, ANFIS, multiple non-linear regression, multivariate adaptive regression splines, and generalized regression neural network. ANFIS with bell-shaped membership function and ANN were found to be better when compared to other models. In a study, the efficacy of soft computing approaches such as SVM, M5P, and multiple non-linear regression was estimated for the prediction of volumetric oxygen transfer coefficient. The experimental tests were performed on hollow jet aerators with different jet plunging angles i.e., 30°, 45°, and 60°. The results indicated that SVM was the best model among other regression models^43^. In the current work, experiments are carried out to study the aeration efficiency of plunging jets fabricated from acrylic sheets. The hydraulics lab's tilting flume equipment was used for the experiments. As far as authors are aware, significantly less literature is available on jet aeration in open channel water flow. None of the studies utilizes a range of the aforementioned input parameters to investigate the aeration efficiency.

### Null Hypothesis (H_0_):

Input variables considered in the present study such as θ (°), Q (L/s), J_n_ (Number), HR_Jn_ (cm), and *Fr*, do not have effect on output variable, E_20_.

### Alternate Hypothesis (H_A_):

The aforementioned input variables have significant effect on E_20_.

Thus, the study is innovative and highlights the following goals:

- Impact of input parameters such as such as θ (°), Q (L/s), J_n_ (Number), HR_Jn_ (cm), and *Fr*on output variable, E_20_.
- Prediction of E_20_ with various soft computing techniques, ANN, M5P, and RF.
- Sensitivity analysis to ascertain the consequences of each variable onE_20_.

## Soft computing techniques

The following section shows the soft computing techniques that were modelled to predict E_20_ in the current study.

### Artificial neural networks

The first ANN was established in the field of biology, where the structure and function of biological neurons and neural networks served as the inspiration for the design of these computer systems. While "network" in ANN relates to the interrelated framework of such neurons in biological systems, "neural" in ANN pertains to a neuron. An ANN comprises unified artificial neurons set up to resemble the characteristics of natural neurons. These neurons work together to solve a particular problem. The ANN design incorporates many user-defined features that are customized with machine-learning models. For a realistic ANN network, utilise trial and error. The prediction equation is hidden via black-box approaches. Notes show how often the layers provide data to the network. Epochs are training data cycles^44,45^. ANNs have a training period that expands exponentially as dataset size does. one or more hidden layers with computational neurons that improve and transmit the information from the preceding layer, one input layer with a prediction node, and one input layer with neurons representing input variables^46,47^. A network comprising biases, a sigmoid layer, and a linear output layer by an approximate finitely discontinuous function^48^.

### Random forest

There is considerable interest in machine learning research concerning ensemble learning methods for generating many classifiers and combining their results. Many ensemble methods are widely used, including boosting bagging and, more recently, random forest (RF)^49^. The RF approach converts input vectors into a planned work of tree predictors using random input samples. Breiman^50^ devised the random forest technique, and later proved to be a highly effective all-purpose characterization and correlation tool. The parameters are selected based on the optimal split, and the technique is hit-or-miss. By capturing a collection of random trees, the RF technique creates random forests^51^. RF functions by combining weak classification trees and makes decisions by a majority vote, combining bagging and random subspace. The number of features will be examined to determine the optimal splitting and the number of decision trees to create (Ntree), in order to properly set splitting for the forest trees^52,53^. In reality, two-thirds of the training data is used to generate every tree. Performance may be calculated using the Out-of-Bag (OOB) data, the part of training samples that were not utilized. Consequently, there are N trees in the random forest regression, where N is the maximum number of trees to be created, which the user may specify to any integer. Each forest's 'n' tree is built using the CART (classification and regression trees) method without pruning. When utilizing different criteria and RF regression, the tree can be allowed to grow to the depth of all new training data. A "Gini" index is utilized to measure the degree of inaccuracy in the parameters compared to the result before selecting a training set of parameters to build specific trees. Compared to a single regression tree, a regression forest is less predictive. The training dataset is critical when a single tree splits into a single criterion. Minor adjustments to the dataset and splitting criteria may prime different tree topologies, resulting in different conclusions. RF models categorize the variables according to their relevance to create the best RF model^50^.

### M5P

Quinlan^54^ developed the M5P algorithm, which has the advantage of being able to handle large data with many traits efficiently. Additionally, they can handle inaccurate information without introducing any uncertainty. This tree approach classifies or divides diverse data areas into several sub-spaces at the terminal area, then enforces a linear regression on each multivariate linear regression model sub-location. The M5P tree is constructed in two steps. A splitting method is used to build a decision tree in the initial stage. The branching criteria produced by the M5P tree model approach are based on the behavioural class labels that approach a branch to measure the inaccuracy and the predicted decrease in error due to evaluating each characteristic at that node. The primary tree model may be produced owing to the separation criteria's ability to anticipate the standard deviations of class values extending to nodes. The data is cleaner because this method constructs linear functions at each node and calculates the predicted errors at the node using the standard deviation technique. For this standard deviation reduction formula (SDR) is given as:

5 $$SDR=sd\left(N\right)-\frac{{\sum }_{i=1}^{x}\left|{N}_{i}\right|}{\left|N\right|}*sd\left(N\right),$$

where ‘$N$’ is sample size, ${N}_{i}$ is the ${i}^{th}$ sample and ‘$sd$’ is the standard deviation.

The tree is pruned in the second stage. The final stretch excludes the marginalized branches (terminal sections) to ensure strong prediction performance. This procedure comprises selecting the components that should be trimmed based on a criterion. After being trimmed, the fresh leaves are located using the arrangement of data used in the learning procedure. This smoothing method then typically results in predictions that are better. In this and subsequent steps, a regularization technique is used to solve for irregularities in surrounding linear models in the leaves of the tree.

### Performance evaluation

#### Correlation coefficient (CC)

One of the most often used and reported statistical techniques is the correlation coefficient (CC), also referred to as Pearson's correlation. This statistical technique is employed to estimate how closely a linear connection is related. It has a value between − 1 and + 1. The correlation is shown by the numbers − 1 for a negative correlation, + 1 for a positive correlation, and 0 for no correlation.

6 $$CC=\frac{\sum_{i=1}^{N}\left({k}_{i}-\overline{k }\right)\left({l}_{i}-\overline{l }\right)}{\sqrt{\sum_{i=1}^{N}{\left({k}_{i}-\overline{k }\right)}^{2}}\sqrt{\sum_{i=1}^{N}{\left({l}_{i}-\overline{l }\right)}^{2}}},$$

where, (${k}_{i}$) represents predicted value ($\overline{k })$ represents mean of predicted valueand (${l}_{i}$) represents observed value.

#### Root mean square error

The sample standard deviation for the variations between real data ($l$) and projected values (${k}_{i}$), is represented by the RMSE, where "$N$" is the number of observations. Normal distribution errors are described by RMSE.

7 $${\text{RMSE}}=\sqrt{\frac{1}{N}\sum_{i=1}^{N}{\left(l-{k}_{i}\right)}^{2}}.$$

#### Mean absolute error

To estimate how well a prediction fits actual results, the MAE is utilised. It is assigning each error the same weight. The uniformly distributed errors are described by MAE.

8 $${\text{MAE}}\hspace{0.17em}=\frac{1}{N}\sum_{i=1}^{N}\left|{l}_{i}-{k}_{i}\right|.$$

## Methodology

**Figure 1:**
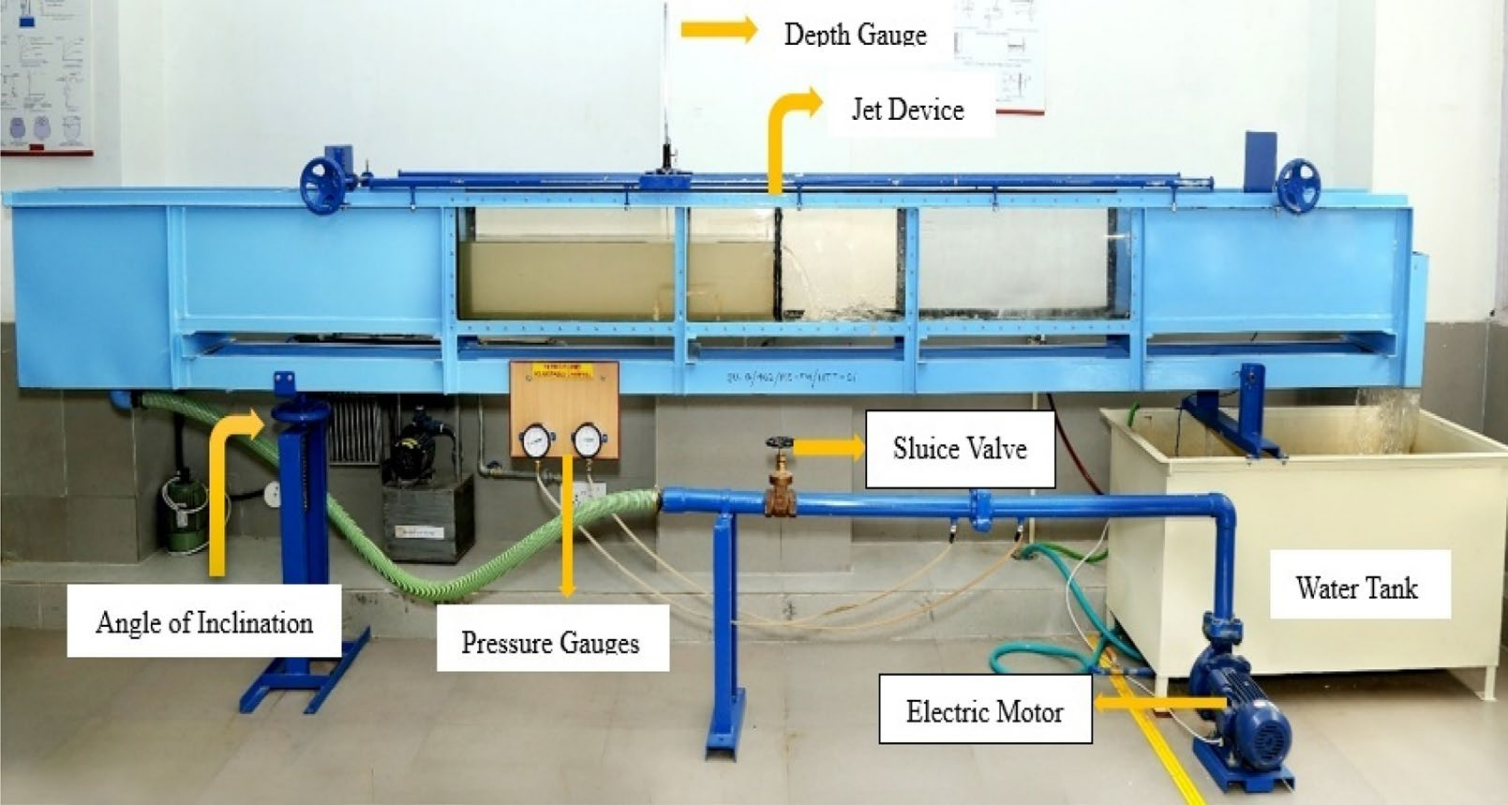
Tilting flume equipment experimental setup.

**Figure 2:**
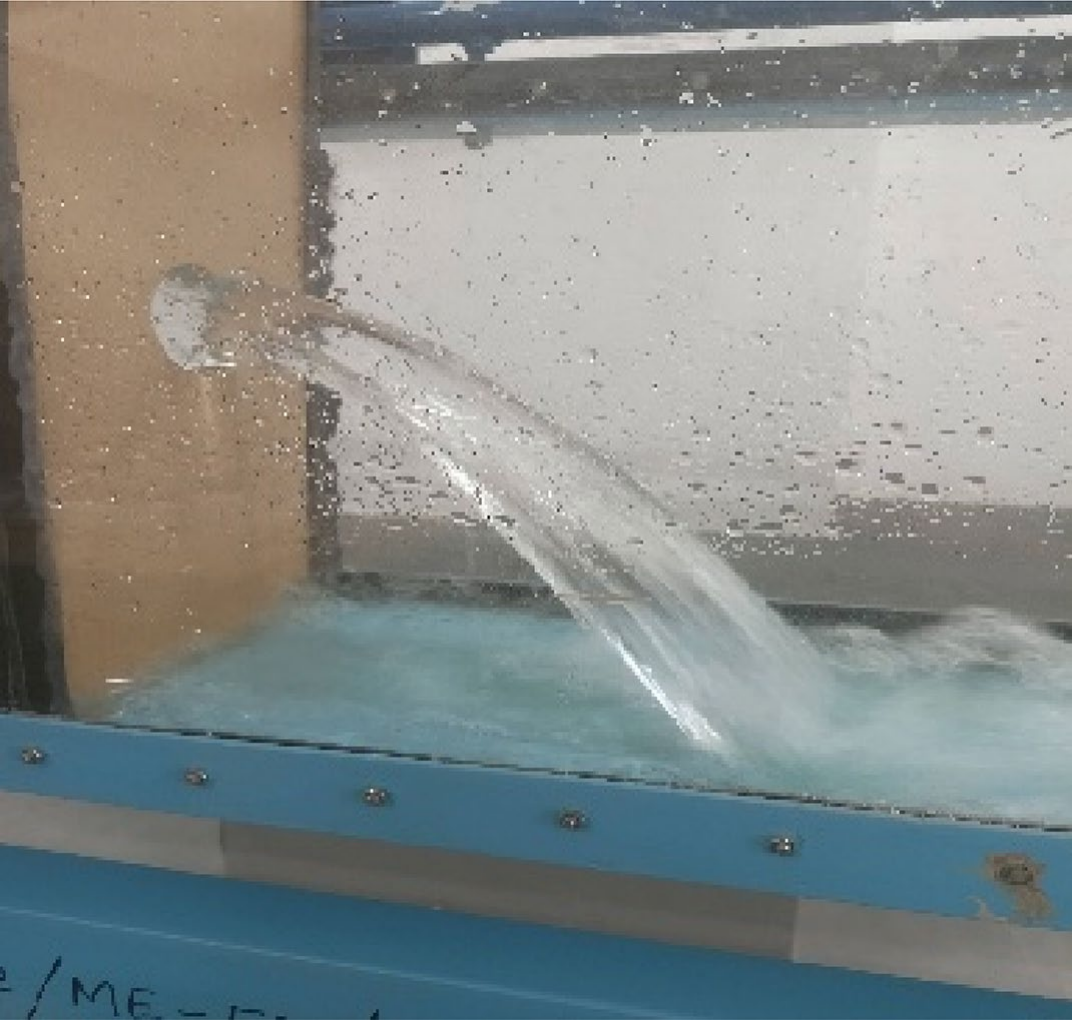
Plunging water jet with J_n_ = 1.

**Figure 3:**
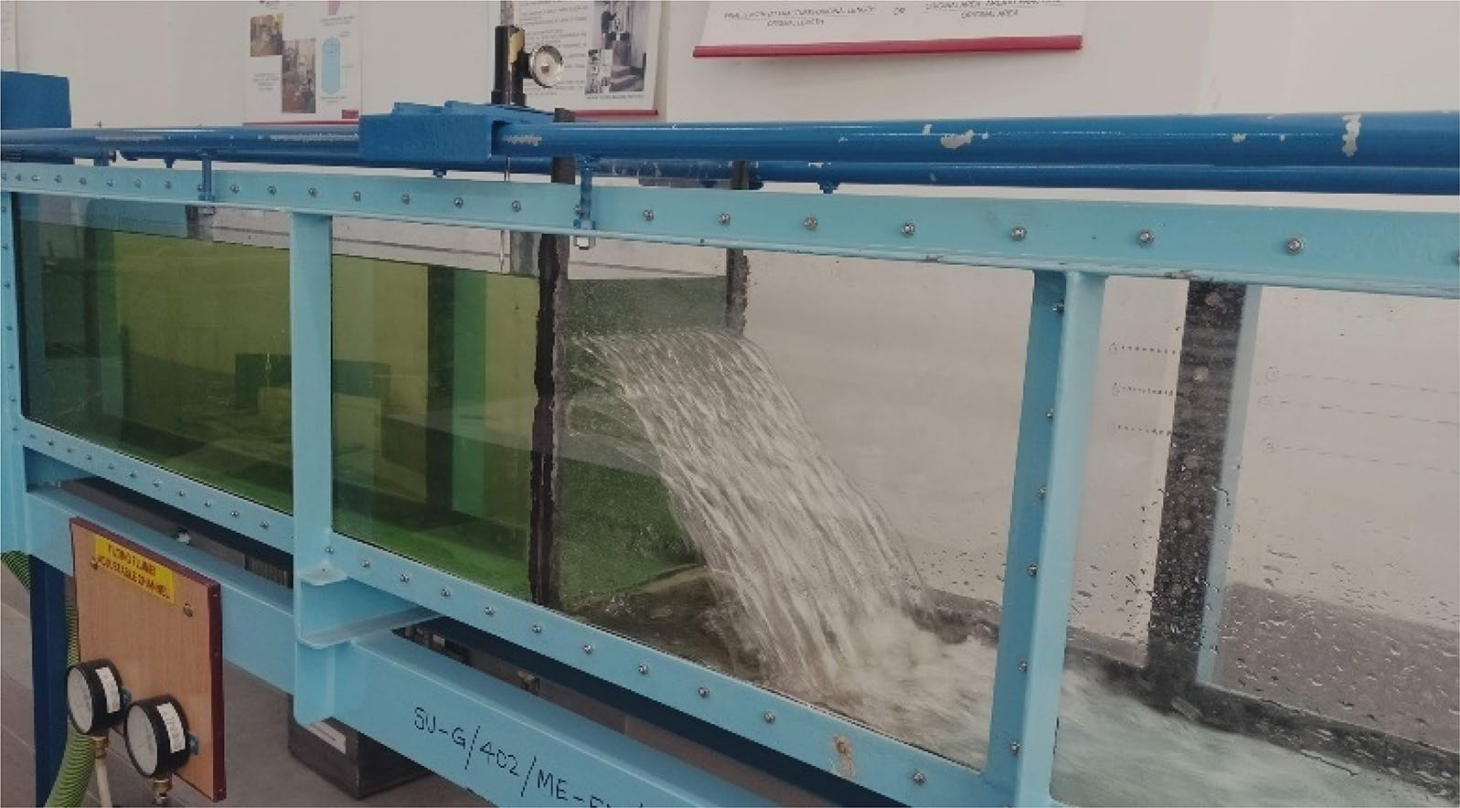
Plunging water jet with J_n_ = 64.

Figures 1, 2 and 3, show the experimental tests for the current investigation were carried out in a tilting flume with dimensions of 45 × 25 × 500 cm. A 2 HP electric motor was used to circulate the water in the flume. Seven interchangeable acrylic sheets with 1, 2, 4, 8, 16, 32, and 64 jets were included as the aeration device (Table 1).Each screen was evaluated for Q values of 3.41L/s, 3.84L/s, 4.75L/s. It has been found that Q is in the range of 0.1L/s–4.69L/s in the studied literature^55–57^.

**Table 1:** Jets configuration (plate dimensions = 45 × 25 cm).

Jets configuration	J_n_	Diameter of each jet (cm)
	1	6.255
	2	4.423
	4	3.127
	8	2.211
	16	1.563
	32	1.105
	64	0.781

**Figure 4:**
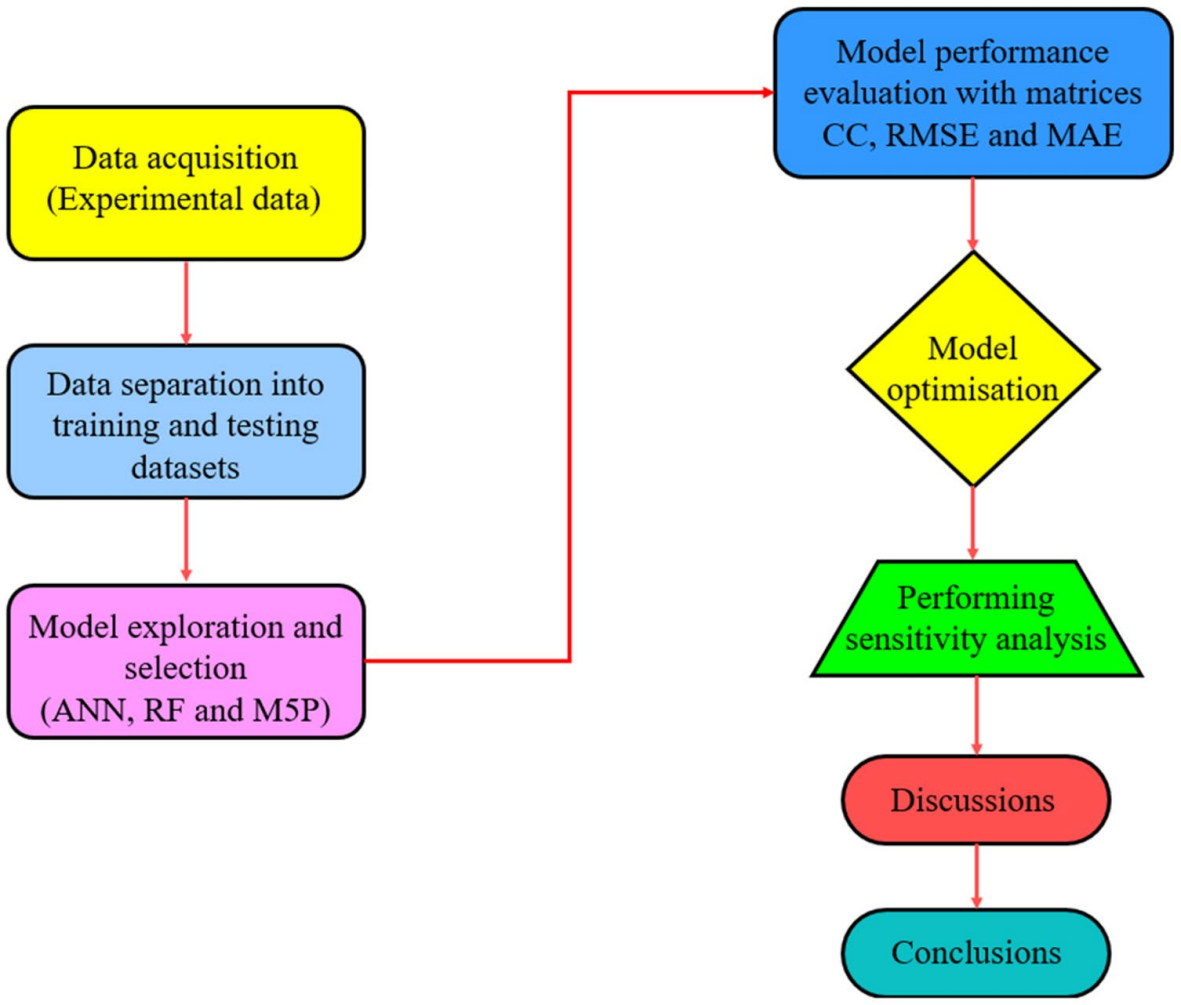
Representation of methodology.

The discharge in the field examples was found to be in between 1L/s and 6L/s in case of aquifer systems in Bengaluru^58^, and 1.1L/s-8L/s as recommended by WATEX^59^. The values of θ considered in the current study are 0°, 1.5°, and 3°. Every acrylic sheet was positioned within the flume and adjusted such that water only enters the pool downstream through jet holes. In order to deoxygenate the tank water before the tests could begin; Sodium Sulphite (Na_2_SO_3_) and a catalyst called Cobalt Chloride (CoCl_2_) were introduced in the water tank. Using the azide modification method^60^, the initial concentration of dissolved oxygen (${C}_{up}$) was found in a sample of oxygen-depleted water that was taken upstream of the jet device. The next step was aeration for a predetermined period (t = 2 min). Then sample of oxygenated water was taken to estimate the concentration of dissolved oxygen in the water downstream (${C}_{down}$) of the aeration device after time ‘t’. A lab thermometer was used to monitor the water's temperature during the experiments. Equations (2), (3), (4) were then used to get the value of E_20_. The input and output data for the 63 experiments is listed in Table S1 (supplementary data). Further three soft techniques; ANN, M5P, and RF were used to predict E_20_. Out of the total of 63 experimentally recorded readings, the 42 readings were randomly chosen for training dataset, rest 21 readings were considered for testing dataset. The traits of both collections of datasets are shown in Table 2. It shows the characteristics of data such as mean, median, standard deviation etc. to check the comparison of the training and testing dataset. These have been used to validate the testing dataset. A representation of the procedure is shown in Fig. 4.

**Table 2:** Statistics of dataset.

Training dataset						
	θ (°)	Q (L/s)	J_n_ (Number)	HR_Jn_ (cm)	*Fr*	E_20_
Mean	1.500	3.968	18.143	0.695	2.948	0.183
Median	1.500	3.840	8.000	0.553	2.735	0.193
Std. dev	1.240	0.560	21.494	0.464	1.110	0.063
Kurtosis	− 1.538	− 1.416	0.470	− 0.666	− 0.792	− 0.741
Skewness	0.000	0.522	1.330	0.764	0.438	− 0.107
Minimum	0.000	3.410	1.000	0.195	1.416	0.070
Maximum	3.000	4.750	64.000	1.564	5.577	0.322

Testing dataset						
	θ (°)	Q (L/s)	J_n_ (Number)	HR_Jn_ (cm)	*Fr*	E_20_
Mean	1.500	4.064	18.143	0.695	2.995	0.186
Median	1.500	3.840	8.000	0.553	3.188	0.187
Std. dev	1.255	0.578	21.761	0.469	1.144	0.064
Kurtosis	− 1.579	− 1.762	0.706	− 0.587	0.624	− 0.981
Skewness	0.000	0.237	1.383	0.794	1.036	− 0.062
Minimum	0.000	3.410	1.000	0.195	1.896	0.093
Maximum	3.000	4.750	64.000	1.564	5.577	0.298

## Experimental results

### Effect of number of jets

**Figure 5:**
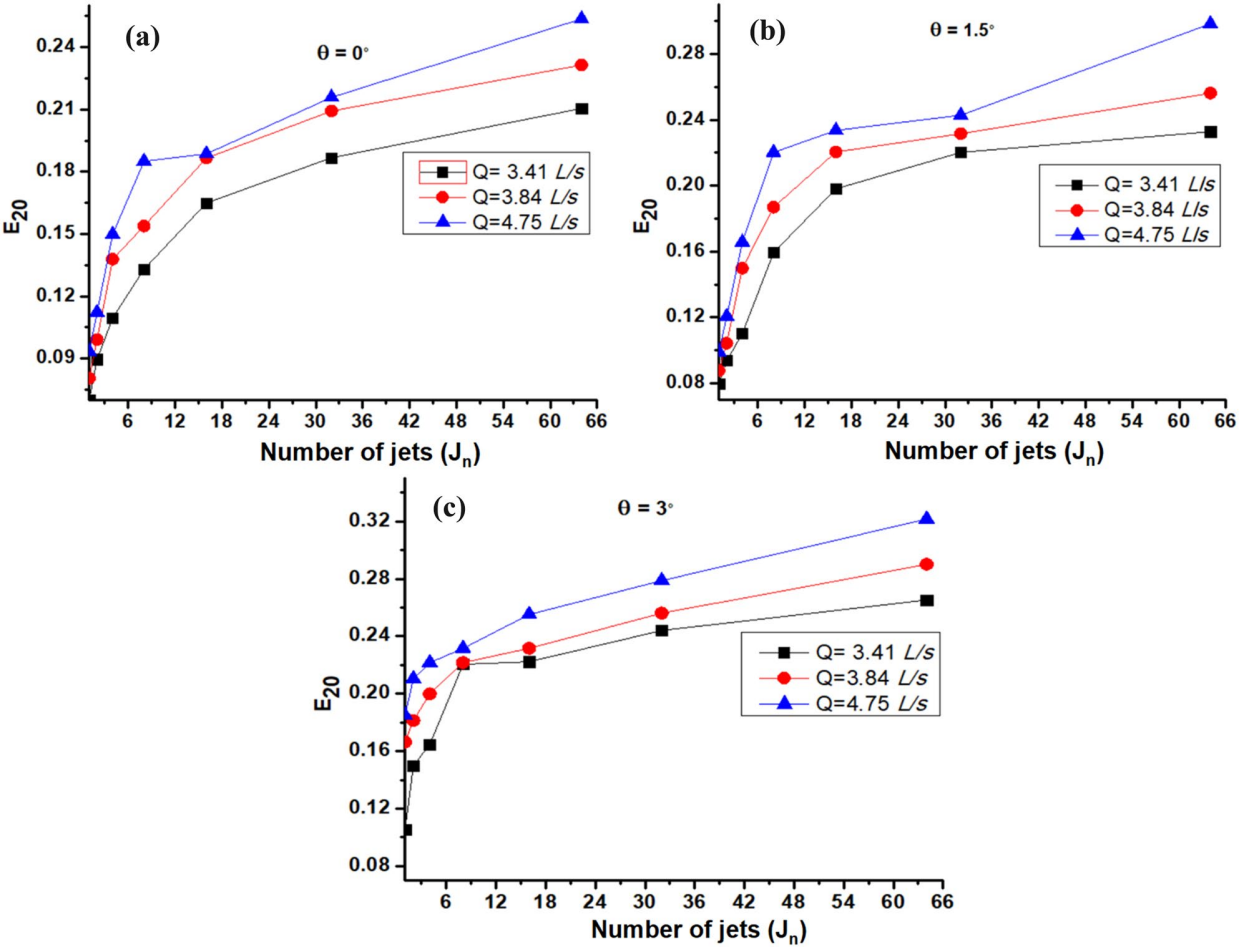
Effect of J_n_ on E_20_ at θ (**a**) 0° (**b**) 1.5° and (**c**) 3°.

Figure 5 demonstrates the impact of the number of jets (J_n_) on E_20_ at angles of inclination (θ) 0° (Fig. 5a), 1.5° (Fig. 5b), and 3° (Fig. 5c). The increase in E_20_ that occurs as J_n_ rises may be seen in Fig. 5. At angle of inclination 0°, J_n_ = 64 showed the largest increase, ranging from 0.21–0.25. With J_n_ = 64, aeration increased to 0.22–0.29 at angle of inclination of 1.5°. At angle of inclination 3º, J_n_ = 64 gives maximum aeration between 0.26 and 0.32.To sum up, the jet device with the maximum number of jets i.e., J_n_ = 64 provides E_20_ from 0.21 to 0.32 from angle of inclination 0º to 3º. This increase in E_20_ with an increase in the J_n_ for multiple plunging jets could be credited to more air/oxygen being present as a result of the increasing surface area of many jets in contact with the atmosphere becoming entrained.

### Effect of discharge

The effect of discharge (Q) was also observed on E_20_, as shown in Table 3. It was found that the E_20_ increase is 33.4% and 20.54%;24.08% and 28.11%; 76.02% and 21.28% for J_n_ = 1 and J_n_ = 64, respectively, at angle of inclination (θ = 0°, 1.5°, and 3°) when Q is increased from 3.41L/s to 4.75L/s. It was found that higher Q can contribute to higher E_20_. The E_20_ increase was found in the range of 20–76% in plunging jets for J_n_ = 1 and 64. The increased number of jets and greater discharge provide a larger air–water contact area, which increases turbulence. This increased turbulence can be linked to an increase in E_20_.As the discharge is increased from 3.41L/s, to 4.75L/s, the jets acquire sufficient kinetic energy to pierce deeper into the tank, and more oxygen is pushed into the pool as a result of a larger air–water contact area.

**Table 3:** Values of E_20_ for different discharge, angle of inclination, and jet numbers.

Q (L/s)	J_n_ (Number)	θ (°)		
		0(°)	1.5(°)	3(°)
3.41	1	0.070	0.080	0.105
	64	0.210	0.233	0.265
3.84	1	0.080	0.088	0.166
	64	0.231	0.256	0.290
4.75	1	0.093	0.099	0.185
	64	0.254	0.298	0.322

### Effect of angle of inclination

Table 3 shows the effect of angle of inclination (θ) on E_20_. It was observed that a higher angle of inclination contributed to a higher E_20_. The increase in E_20_ between θ from 0° and 3°was found to be higher than 25% varying in jet number (J_n_) 1 and 64in plunging jets. The increased air–water contact area caused by the multiple jet holes and turbulence at a higher angle of inclination, as well as the increased velocity of the jet, are all responsible for the increase in E_20_ with θ.

### Effect of Froude number

**Figure 6:**
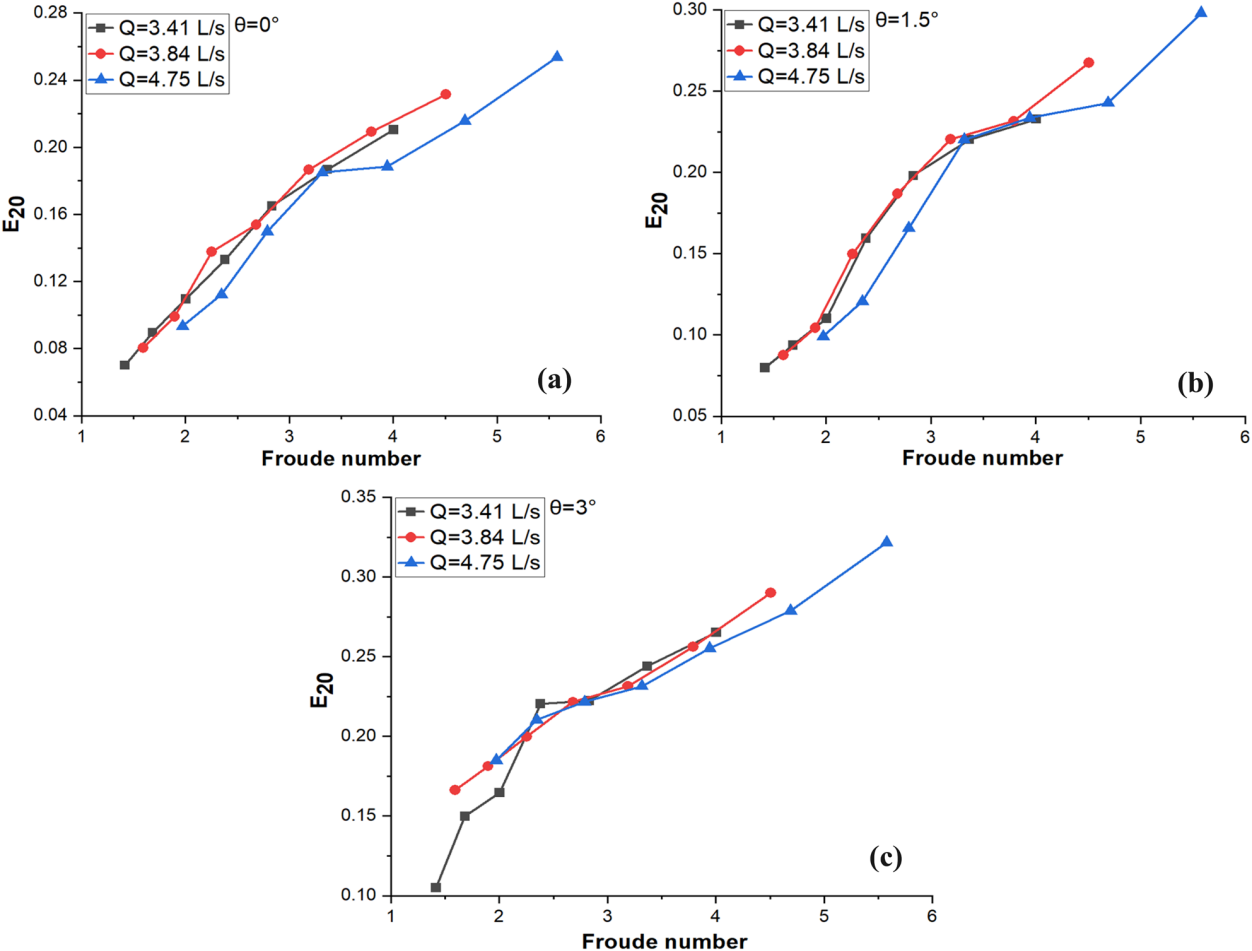
Effect of *Fr* on E_20_ at θ (**a**) 0° (**b**) 1.5° and (**c**) 3°.

The impact of the Froude number of each jet (*Fr*) on the E_20_ at different angle of inclination and discharge rate is illustrated in Fig. 6a–c. The discharge rate and jet area affect the *Fr* and is determined using the following Eq. (9):

9 $$Fr= \frac{v}{\sqrt{g\times {D}_{{J}_{n}}}}; {\mathrm{ J}}_{{\text{n}}}=1, 2, 4, 8, 16, 32, 64.$$

Here, $v$ is the average velocity measured in the downstream of the plunging jets after bubble formations (cm/s), and g denotes accelerated gravity (cm/s^2^). While D_Jn_ is the diameter of each jet determined using the following equation:

10 $${{\text{D}}}_{{{\text{J}}}_{{\text{n}}}}=2\sqrt{\frac{{\text{Jet}}\hspace{0.33em}{\text{area}}}{\uppi \times {{\text{J}}}_{{\text{n}}}}};\hspace{1em}{{\text{J}}}_{{\text{n}}}=1, 2, 4, 8, 16, 32, 64.$$

The various parameters used in the calculation are listed in Table 4. The total jet area is 30.75cm^2^, so the ${{\text{D}}}_{{{\text{J}}}_{{\text{n}}}}$ reduces with increased J_n_ values. The *Fr* value found to increase with increase in Q and decrease in ${{\text{D}}}_{{{\text{J}}}_{{\text{n}}}}$.

**Table 4:** Results of parameters used in *Fr* calculation.

J_n_	Cross- sectional area of each jet (cm^2^)	${{\text{D}}}_{{{\text{J}}}_{{\text{n}}}}$	*Fr*		
			${\text{Q}}=3.41{\text{L}}/{\text{s}}$ $v=110.89 cm/s$	${\text{Q}}=3.84{\text{L}}/{\text{s}}$ $v=124.87 cm/s$	${\text{Q}}=4.75{\text{L}}/{\text{s}}$ $v=154.47 cm/s$
1	30.75	6.2559	1.41	1.59	1.97
2	15.375	4.4236	1.68	1.89	2.34
4	7.687	3.1280	2.00	2.25	2.78
8	3.843	2.2118	2.38	2.68	3.31
16	1.921	1.5640	2.83	3.18	3.94
32	0.960	1.1059	3.36	3.79	4.68
64	0.480	0.7820	4.00	4.50	5.57

In Fig. 6a–c, it is noted that E_20_rises with rise in *Fr*. The E_20_ also noted an increase with an increase in Q value from 3.41 to 4.75 L/s and θ from 0° to 3°. This is due to higher fluid velocity and increased inclination angle of the slope that affect the *Fr* of the fluid. As the fluid velocity increases, the *Fr* increases, indicating that the effects of inertia become more dominant. Similarly, increasing the inclination angle of the slope also leads to an increase in the *Fr*. Furthermore; the E_20_ of a system is affected by the *Fr,* as it influences the rate of air entrainment. When the *Fr* is low (*Fr* < 1), the flow is considered subcritical, and there is a tendency of air bubbles to rise slowly and follow the flow, resulting in less air entrainment in the fluid. Conversely, with high Froude numbers (*Fr* > 1), the flow is considered supercritical which cause air bubbles to break up into smaller ones due to high turbulence in water pool, leading to increased air–water interfacial area and thus enhanced air entrainment rate. Therefore, to attain maximum E_20_, an optimal *Fr* must be achieved.

### Effect of hydraulic radius of jets

The cumulative hydraulic radius (HR) is extremely important for fluid mechanics in an open channel. It is determined using the following equation.

11 $${\text{HR}}={{\text{HR}}}_{{{\text{J}}}_{{\text{n}}}}\times {{\text{J}}}_{{\text{n}}};{\mathrm{ J}}_{{\text{n}}}=1, 2, 4, 8, 16, 32, 64,$$

12 $${{\text{HR}}}_{{{\text{J}}}_{{\text{n}}}}=\frac{{\text{Jet}}\hspace{0.33em}{\text{area}}}{\uppi {\times {{\text{D}}}_{{{\text{J}}}_{{\text{n}}}}\times {\text{J}}}_{{\text{n}}}}.$$

**Figure 7:**
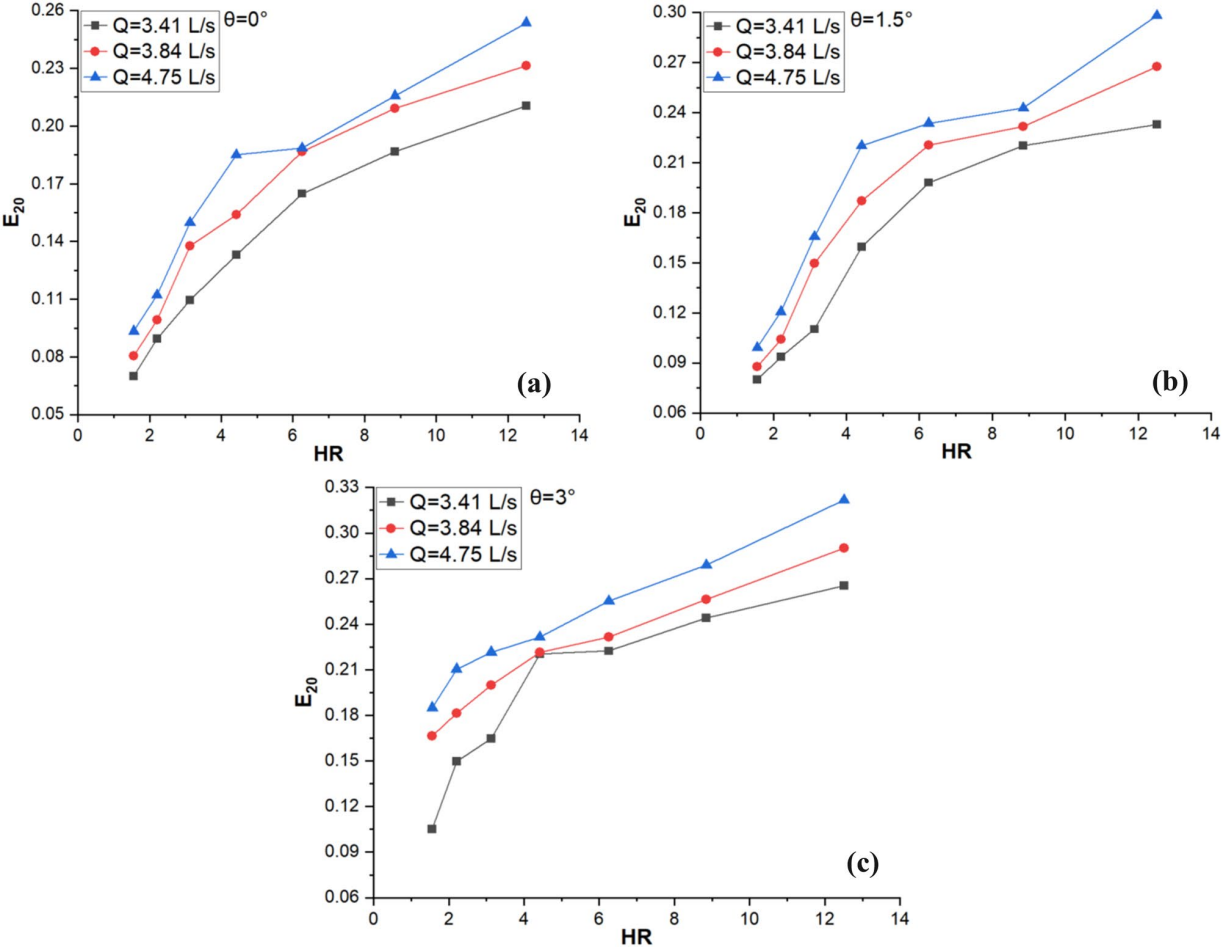
Effect of HR on E_20_at θ (**a**) 0° (**b**) 1.5° and (**c**) 3°.

The impact of HR on theE_20_ at different discharge rates and angle of inclination is illustrated in Fig. 7a–c and it shows that there is increasing trend between HR and the E_20_. The E_20_is also noted to increase with an increase in θ from 0° to 3° and the Q value from 3.41 to 4.75 L/s. Wetted perimeter decreases with increasing HR, indicating that a smaller amount of water is in proximity to the channel portion which lowers the resistance to flow and enables more discharge to pass through it, resulting in increased E_20_.

## Statistical analysis

### Post hoc test

Table 5 shows ANOVA results among J_n_ and E_20_ values of plunging jets for the present study.

**Table 5:** ANOVA results with J_n_ and E_20_.

ANOVA					
E_20_					
	Sum of squares	df	Mean square	F test (F)	Significance value
Between groups	0.171	6	0.029	22.372	0.000
Within groups	0.071	56	0.001		
Total	0.243	62			

It is observed that the F and significance values are 22.372 and 0.00 (less than 0.05) respectively. Thus, the results are relevant with respect to the null hypothesis to be rejected. A Post-hoc analysis has been carried out in order to investigate the significance of differences between pairs of group means. The dependent variable considered for carrying out the post-hoc test was E_20_, and the independent variable was J_n_. In Table 6, the J_n_ = 1 (single jet) was considered as control and the other multiple jets were found to have substantial differences in the mean. It was observed that significant value for J_n_ = 2 and J_n_ = 4 was higher than 0.05. Therefore, these jets are insignificant. It was also found that J_n_ = 8 to J_n_ = 64, have a significance value less than 0.05 hence they have significant impact on E_20_. Another observation from this table can be drawn that J_n_ = 64 has the highest mean difference, and thus it provides the maximum E_20_.

**Table 6:** Post-hocTukey’s analysis results for single and multiple jets.

Multiple comparisons						
Dependent variable: E_20_						
Tukey’s HSD						
(I) J_n_	(J) J_n_	Mean difference (I-J)	Standard error	Significance value	95% confidence interval	
					Lower bound	Upper bound
1	2	− 0.0215	0.0168	0.858	− 0.0730	0.0299
	4	− 0.0491	0.0168	0.071	− 0.1005	0.0023
	8	− 0.0828	0.0168	0.000	− 0.1342	− 0.0313
	16	− 0.1038	0.0168	0.000	− 0.1552	− 0.0523
	32	− 0.1242	0.0168	0.000	− 0.1757	− 0.0728
	64	− 0.1560	0.0168	0.000	− 0.2075	− 0.1045

The F value is ratio of variances of two data sets whereas degrees of freedom represent the interval group between two input parameters. In a multi-group comparison, it exhibits the statistical significance of difference in group means.

The F-value of 22.372 showed that the ratio of variance of one dataset was 22.372 times of the second dataset, implying that the means of these two variances were not equal and hence null hypothesis was rejected and alternate hypothesis is accepted. The fact was also verified by obtaining the significance value as 0.000 which is p-value which meant that recognised values obtained were significantly distinct from the sample population value which was initially hypothesised.

The p-value less than 0.05 is responsible for rejecting null hypothesis which is confirmed by the F-test value. The input parameter number of jets (J_n_) has 7 inputs i.e., 1, 2, 4, 8, 16, 32 and 64 and degree of freedom (df) in this case is 7–1 = 6.The value of F-critical obtained from the F-table with degree of freedom (df) = 6 was found to be 5.9874 at confidence level 0.05. Since the F critical (5.9874) is less than F-calculated (22.372), the null hypothesis is rejected which showed that number of jets (J_n_) affected the E_20_ significantly.

The column 1 and 2 showed the No. of jets (J_n_) wherein column 1 is the reference column and performance of No. of jets in column 2 is compared with No of jets in column 1 by exhibiting significance (p) value which is required to be less than 0.05 for Null hypothesis to be rejected. The mean difference (I-J) showed the difference of E_20_ values for I and J columns. The standard error showed the error between observed value and mean values. The significance value showed the p-value which is significant if it is less than 0.05. The confidence level of 95% interval showed the values of mean difference (I-J) felled in the interval of lower and upper bound interval.

### Linear regression analysis

Table 7 shows the regression statistics for which R (correlation coefficient) and R^2^ values are close to 1, which testifies the model to be satisfactory. Table 8 shows coefficient results with input parameters θ, Q, J_n_, HR_Jn_, and *Fr* based on which the model (Eq. (13)) was generated.

**Table 7:** Regression statistics for jets.

R	R^2^	Adjusted R^2^	Standard error of the estimate
0.974	0.949	0.945	0.0147253

**Table 8:** Linear regression model.

Model: (Input parameters)	Un-standardized coefficients		Standardized coefficients	T test (t)	Significance value
	Regression coefficient (B)	Standard error	Beta		
(Constant)	0.070	0.015		4.645	0.000
θ	0.022	0.002	0.427	14.280	0.000
Q	0.016	0.008	0.141	1.840	0.071
J_n_	0.000	0.000	0.146	1.333	0.188
HR_Jn_	− 0.059	0.013	− 0.436	− 4.631	0.000
*Fr*	0.018	0.011	0.316	1.685	0.097

The Table 8 showed the values of regression coefficient which represented the equation of regression with input parameters for the output parameter E_20_. Standard error gave the values with respect to standard deviation for regression line. The standard coefficients were the coefficients for regression function with constant value as 0. The T- test is the parametric test for comparing means of two groups.

The equation generated with help of the Table 8 is given as under:-

13 $${\text{E}}_{{{2}0}} \, = \,0.0{22}(\theta )\, + \,0.0{16}\left( {\text{Q}} \right)\, + \,0.000\left( {{\text{J}}_{{\text{n}}} } \right)\, + \,0.0{18}\left( {Fr} \right) \, - 0.0{59}\left( {{\text{HR}}_{{{\text{Jn}}}} } \right)\, + \,0.0{7}0.$$

## Computational analysis and results

### Assessment of ANN model

**Figure 8:**
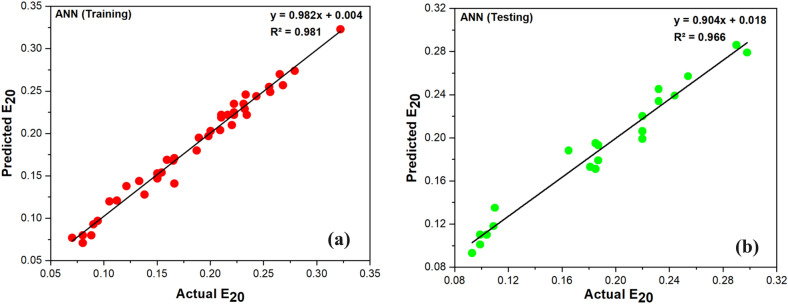
Actual and predicted value of E_20_ using ANN (**a**) Training, (**b**) Testing.

For the current study, ANN results were obtained from WEKA software. Up until the best outcomes were attained, many ANN architectures were tested. It can be tricky to select ANN’s defined functions to get the optimized model, such as hidden nodes, learning rate, and network geometry. Since ANNs only have one hidden layer during training, finding the ideal network geometry is obtained by hit-and-trial. The hidden layer count in this study is 10, the learning rate is 0.2, the momentum is 0.1, and the training time is 550. The ANN model's actual and predicted values for E_20_ during the training and testing phases are shown in Fig. 8. Since the majority of the points in Fig. 8 are fairly close to the tread line, the ANN-based model is appropriate for forecasting E_20_. The outcomes demonstrate a greater consistency between real and anticipated values. The statistical values for each model created for the current investigation are also shown in Table 9. It is found that ANN is the best-predicted model with the highest CC value of 0.9823 in the testing stage and errors, i.e., MAE value of 0.0098 and RMSE value of 0.0123.

**Table 9:** Performances of ANN, M5P and RF model.

	Training dataset			Testing dataset		
	ANN	M5P	RF	ANN	M5P	RF
CC	0.9908	0.9765	0.9928	0.9823	0.9728	0.9682
MAE	0.0067	0.0104	0.0066	0.0098	0.0115	0.0136
RMSE	0.0085	0.0133	0.0082	0.0123	0.0145	0.0163

### Assessment of M5P model

**Figure 9:**
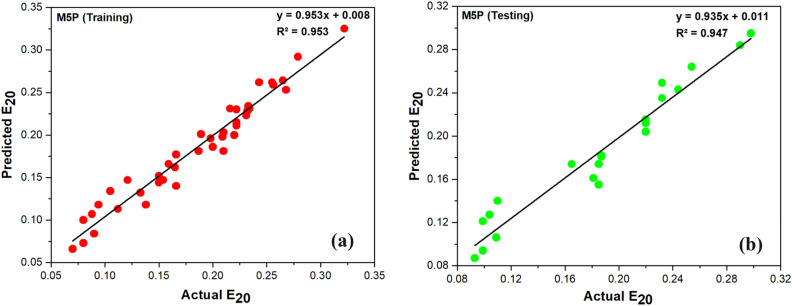
Actual and predicted value of E_20_ using M5P (**a**) Training, (**b**) Testing.

The M5P model generated for this study is used to predict E_20_.The M5P model was developed and validated using the testing and training datasets. In this study, the M5P was trained with a batch size of 100 and a leaf node instance limit of 4. Figure 9a and b show the observations of M5P. The accuracy of a model may be evaluated by comparing the observed data to the predicted value of the slope of the regression line (Fig. 9a,b). Moreover, Table 9 shows the fair result obtained from the M5P model with agreeable CC values in the model development and implementing stages of 0.9765 and 0.9728, respectively. Additionally, it is noted that the MAE and RMSE exhibit reduced values during the training phase but experience a modest rise during the testing phase.

### Assessment of RF model

**Figure 10:**
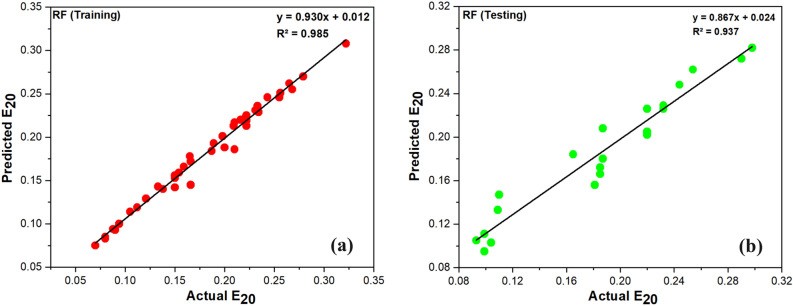
Actual and predicted value of E_20_ using RF (**a**) Training, (**b**) Testing.

WEKA software is also used for the RF-based model's implementation. The RF model is likewise developed using a hit-and-miss approach with some user-defined parameters. Using training and test datasets, the RF model's scattering details for experimental and projected values of E_20_ are shown in Fig. 10. It is evident that each scattering event exhibits the highest level of concordance with the regression line.

### Comparison of soft computing-based models

This section compares the models ANN, M5P, and RF that are used in the current study to predict E_20_. To assess these models, five input parameters; θ, Q, J_n_, HR_Jn_, and *Fr* were taken into account. Table 9 shows the results of evaluating each developed model against three statistical evaluation criteria.

**Figure 11:**
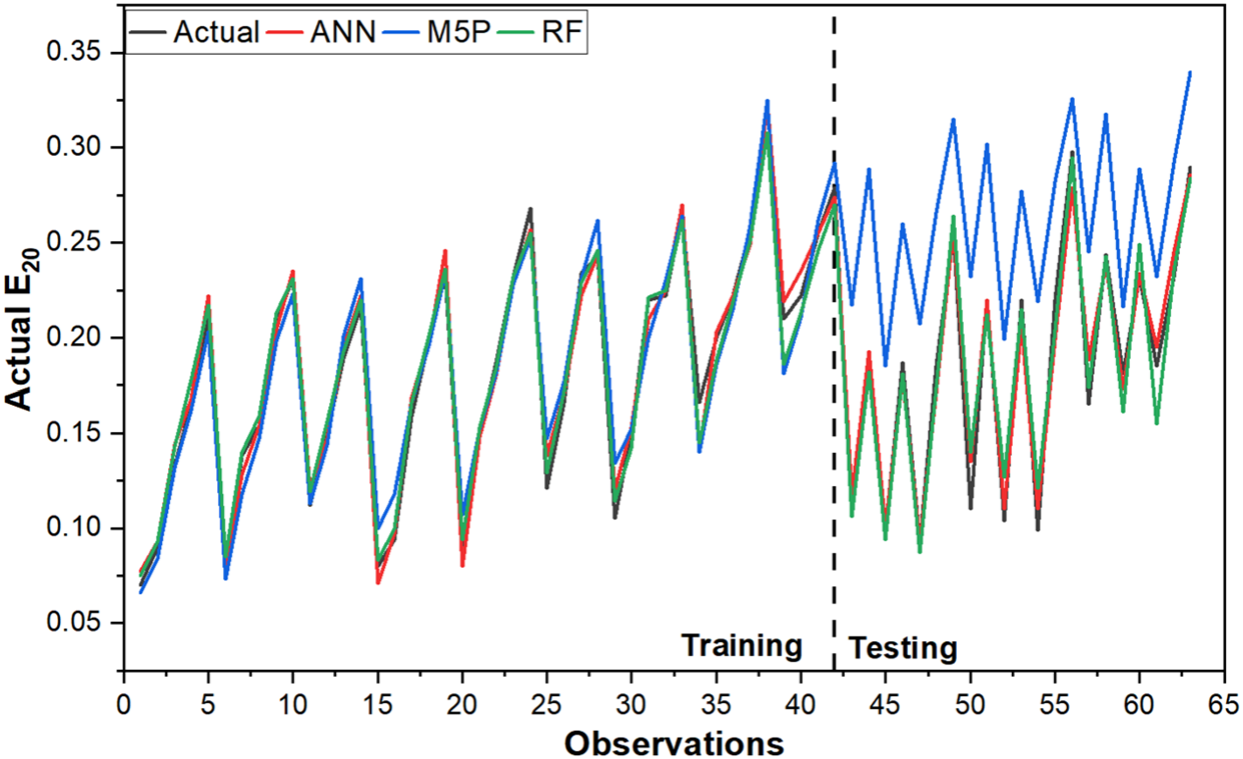
Comparison of ANN, M5P and RF with actual data.

**Figure 12:**
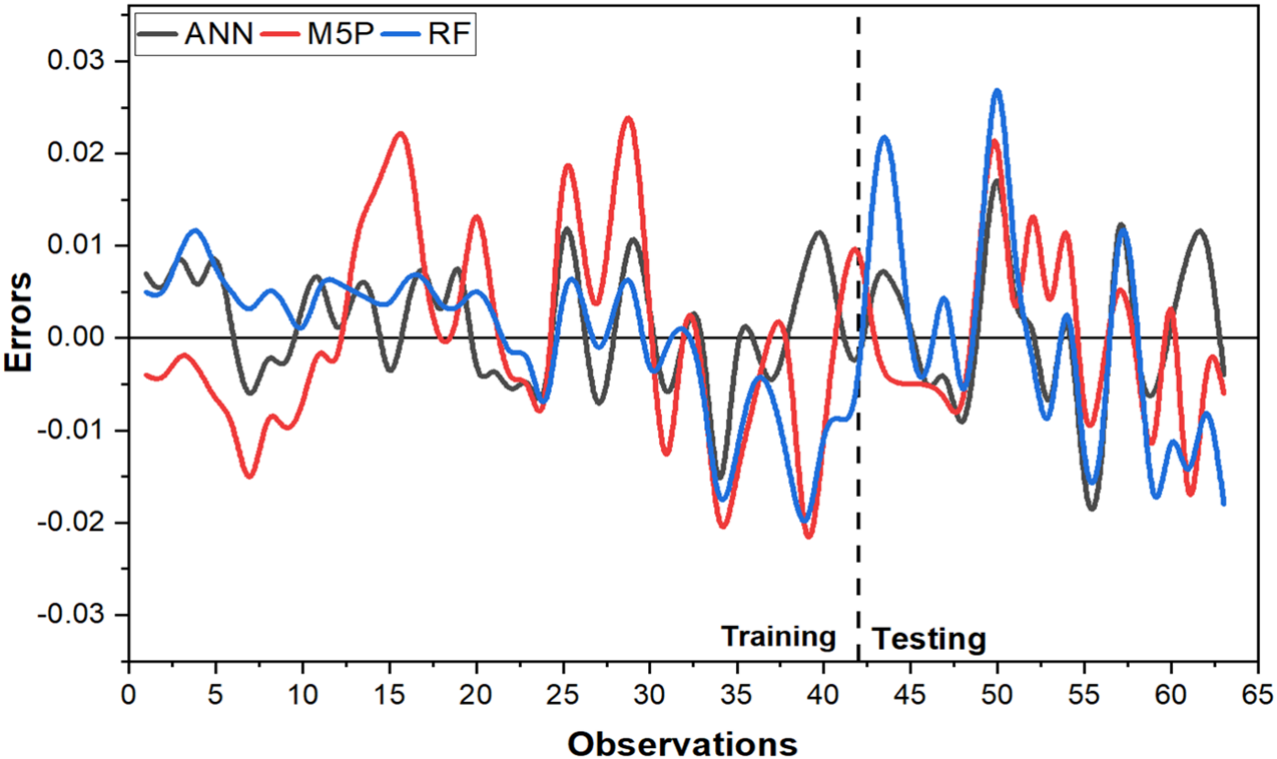
Error values of ANN, M5P and RF in training and testing stage.

**Figure 13:**
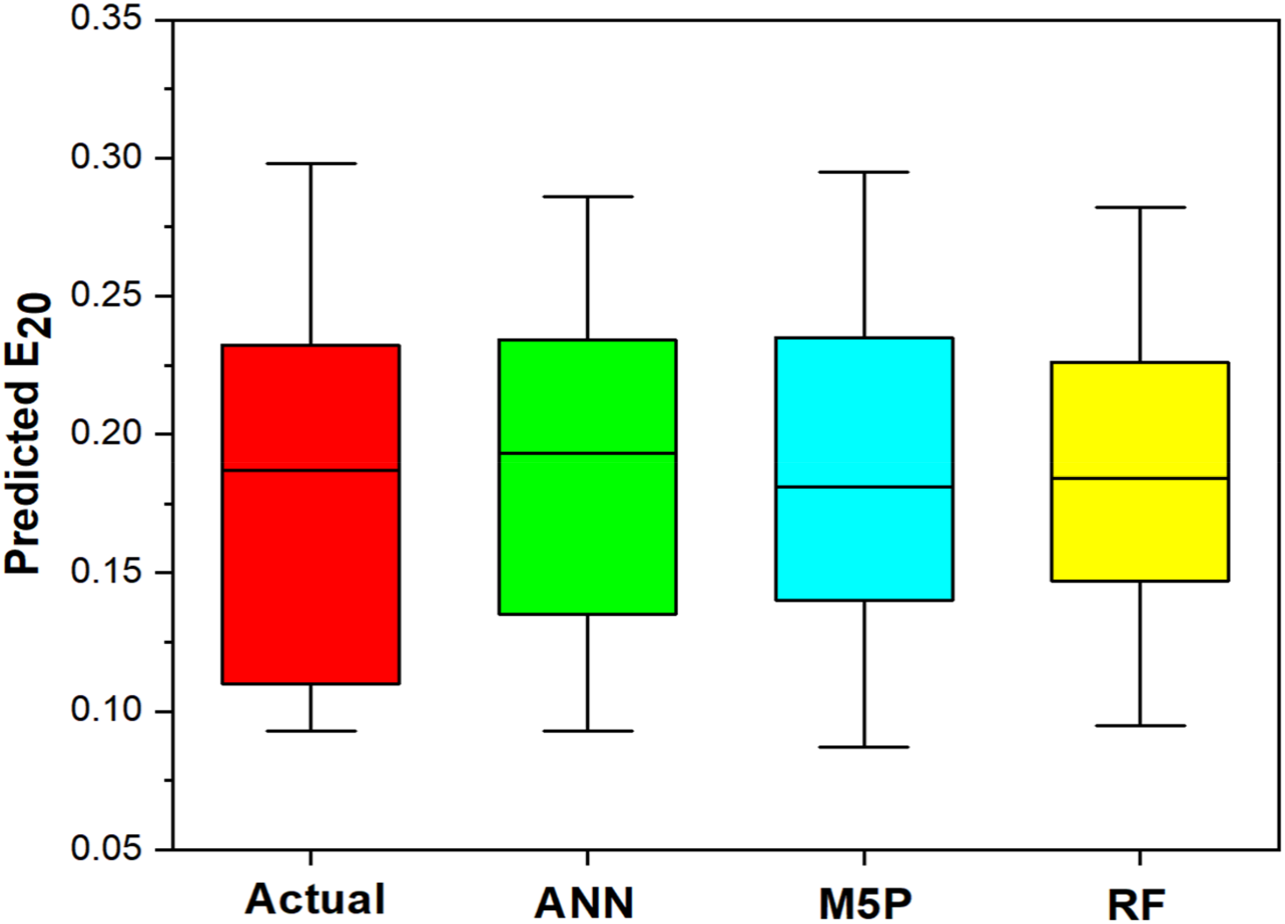
Box plot with actual and soft computing techniques.

The agreement of each model with the data from experiments is shown in Fig. 11, and it is inferred from the graphical representation that the models developed for the study are good at anticipating E_20_. It is also required to evaluate the errors of each model, which are shown in Fig. 12, in order to reach the ultimate outcomes. It indicates that in both the training and testing datasets, RF exhibits more errors than other models. The ANN model demonstrated consistency both before and after training. The box plot of the model outcomes for the testing stage is shown in Fig. 13. The median and maximum values of the actual and ANN models are very close. Actual data has an interquartile (IQR) range of 0.122, while ANN, M5P, and RF have IQRs of 0.099, 0.095, and 0.079, respectively. The difference in the mean between the actual and observed values is minimal in the case of ANN (0.0006).

## Sensitivity analysis

The most important input parameter in predicting the E_20_ of jets in an open channel flow was identified using sensitivity analysis. The outperforming model i.e., ANN was used to carry out sensitivity analysis. A new training dataset was created by gradually eliminating one input parameter, and the results were expressed in terms of CC, MAE, and RMSE. The extent to which the aforementioned evaluation factors changed demonstrates the variable's significance in influencing the E_20_.Findings from Table 10 indicate that, in comparison to other input variables, the angle of inclination of the tilting flume's bed is the most dominant variable and plays a considerable influence in forecasting the E_20_. The tilting flume's bed's angle of inclination increases the horizontal portion of water weight, resulting in higher water velocity. In addition to θ, *Fr* and J_n_ have a higher impact on E_20_.It is well established that aeration efficiency is dependent on θ and J_n_. But when the aforementioned five input parameters are performing collectively in that case the analysis carried out for the sensitivity of each parameter becomes significant to establish their role in achieving E_20_.

**Table 10:** Sensitivity analysis using best fit model.

Combination of input variables	Removed variable	CC	MAE	RMSE
${{\text{E}}}_{20}$= ${\text{f}}$ (${{\text{HR}}}_{{{\text{J}}}_{{\text{n}}}}$,θ, ${{\text{J}}}_{{\text{n}}}$, $Fr$, ${\text{Q}}$)	–	0.9823	0.0098	0.0123
${{\text{E}}}_{20}$= ${\text{f}}$ (${{\text{HR}}}_{{{\text{J}}}_{{\text{n}}}},{{\text{J}}}_{{\text{n}}}$, $Fr$, ${\text{Q}}$)	$\uptheta$	0.8403	0.0315	0.0351
${{\text{E}}}_{20}$= ${\text{f}}$(${{\text{HR}}}_{{{\text{J}}}_{{\text{n}}}}$, θ, ${{\text{J}}}_{{\text{n}}}$,${\text{Q}}$)	$Fr$	0.9751	0.0132	0.0164
${{\text{E}}}_{20}$= ${\text{f}}$(${{\text{HR}}}_{{{\text{J}}}_{{\text{n}}}}$, θ, $Fr$,${\text{Q}}$)	${{\text{J}}}_{{\text{n}}}$	0.9779	0.0102	0.0131
${{\text{E}}}_{20}$= ${\text{f}}$ (θ, ${{\text{J}}}_{{\text{n}}}$, $Fr$, ${\text{Q}}$)	${{\text{HR}}}_{{{\text{J}}}_{{\text{n}}}}$	0.9786	0.0116	0.0134
${{\text{E}}}_{20}$= ${\text{f}}$ (${{\text{HR}}}_{{{\text{J}}}_{{\text{n}}}}$, θ,${{\text{J}}}_{{\text{n}}}$, $Fr$)	${\text{Q}}$	0.979	0.0107	0.0132

## Discussion

In this work, plunging jets with J_n_ values of 1, 2, 4, 8, 16, 32, and 64 and a flow area of 30.75 cm^2^ are made from 7 acrylic sheets. The study examines the E_20_of jets in each sheet in an open channel using parameters likeθ, Q, J_n_, HR_Jn_, and *Fr* as inputs. Each parameter studied significantly affected E_20_. According to the findings, E_20_ rises as J_n_, Q, and θ increase. Several plunging jets transmit oxygen at a rate that is much higher than that of a single jet being plunged into the water pool^28,57^. They also demonstrated that higher discharge results in better oxygenation. The results of the present investigation show that E_20_ increases as J_n_ increases. The results of the current investigation also suggest that E_20_ increases along with discharge. A higher jet impact angle may boost oxygenation by causing more bubbles to interact with the water in the pool as a result of deeper jet penetration and a higher jet angle, which would increase oxygen transfer^61^. According to the current study, aeration gets better as the flume θ rises, reaching a maximum of 0.32 (or 32%) at a 3° angle.

It is well documented in the literature that *Fr* affects turbulence in steady flows of water^62,63^. E_20_ was significantly affected by the *Fr* and the ratio of the water cross-sectional airflow to the duct cross-sectional^64^. Another piece of literature by Puri et al.^65^ demonstrates that an increase in discharge and oxygen transfer has accompanied a rise in *Fr*. The outcomes of the present study also confirm that E_20_ and *Fr* are directly related. It is inferred from the Figs. 5, 6, 7, and Table 3 that E_20_ increases with an increase in input parameters considered in the current study. As the input parameters have an impact on E_20_, therefore, H_0_ must be rejected.

Soft computing, as opposed to conventional computing, approximates complex real-world issues and is tolerant of flaws, ambiguity, partial truth, and assumptions. The human mind serves as an example for soft computing such as fuzzy logic, genetic algorithms, ANN, ML, and expert systems^66^. In the case of severely contaminated water management resources, the prediction of E_20_ is a study that should receive top priority. This work examines the performance of ANN, M5P, and RF soft computing models to predict the jet aeration in an open channel flow. Multiple statistical metrics have been used to measure the efficacy of different models such as CC, MAE, and RMSE. The outcomes demonstrate that ANN is the best predicted model to predict E_20_ while the least-performing model for the given dataset is the RF. According to the current study, all three used models can accurately predict E_20_. However, 10 hidden layers, 550 training time, 0.3 learning rate, and 0.2 momentums have increased the value of CC in the ANN model to 0.9823 over the CC value in M5P and RF to 0.9728 and 0.9682 in the testing stage, respectively, making ANN more effective. However, since M5P and RF both have CC values above 0.95, which is a competent level, their performance cannot be denied. In several research^67,68^, the best predictive model for problems is determined using the ML technique known as ANN. Researchers have also found that depending upon the number of the inputs and computational time Sensitivity analysis was also performed in order to understand the effects of each parameter on E_20_, and the results revealed that the angle of inclination of the tilting flume's input parameter is extremely sensitive to jet aeration in an open channel.

To sum up for the performance of the ANN model over RF model: In the present study, out of the total 63 readings recorded experimentally, 42 were chosen randomly for training dataset, whereas 21 were considered for testing dataset. Random forest may not impart good results for small data sets or low-dimensional data (data with few features). Processing high-dimensional data and feature-missing data are the strengths of random forest^69^. In this case, the small data set of 42 and 21 in training and testing datasets and small dimension of input parameter which were limited to five number i.e., angle of inclination (θ), discharge (Q), number of jets (J_n_), hydraulic radius of each jet (HR_Jn_), and Froude No. (*Fr*) can be the possible reasons for such performance. Whereas the performance of ANN has more manoeuvre capabilities by varying hidden layers, training time, learning rate, momentum rate etc. ANN models provide certain advantages over regression-based models including its capacity to deal with noisy data. ANNs consist of a layer of input nodes and layer of output nodes, connected by one or more layers of hidden nodes. Input layer nodes pass information to hidden layer nodes by firing activation functions, and hidden layer nodes fire or remain dormant depending on the evidence presented. The hidden layers apply weighting functions to the evidence, and when the value of a particular node or set of nodes in the hidden layer reaches some threshold, a value is passed to one or more nodes in the output layer. ANNs can incorporate uncertainties by estimating the likelihood of each output node.

The practical implication of the study is that the DO level in the water has been raised to the level at which the circular geometry of plunging jets is quite helpful in achieving E_20_ to the extent of 32%. This increase can be useful for the cultivation of sericulture, which is progressive aquatic life sustainability. On the other hand, the stakeholders using the oxygenated water can be beneficial for health-related issues. The enriched, oxygenated water can also be congenial to the agricultural and horticultural produce. The oxygenated water is produced by utilising the circular geometrical plunging jets under gravity in open channel flow, for which no electrical power supply is required.

## Conclusions

The current study examines the angle of inclination, number of jets, discharge, Froude number, and hydraulic radius of jets to determine the efficacy of aerating deoxygenated water with a novel form of circular plunging jets produced from acrylic screens. The experimental findings demonstrated that aeration performance in multi-jets is better than that of a single jet. It was found that the E_20_ increase was in the range of 20–76% for J_n_ = 1 to 64 when Q was increased from 3.41L/s to 4.75L/s. It was also found that with an increase of θ from 0° to 3°the increase in E_20_was found to be higher than 25% in the said plunging jets. The post-hoc analysis proved that the number of jets from 8 to 64 significantly affect E_20_. All the parameters, except for the hydraulic radius of each jet, have positive effect on E_20_, according to a developed linear model. Further, E_20_ was predicted using soft computing methods, including ANN, M5P, and RF. It was found that ANN outperformed other applied models with a CC value of 0.9823 in the testing stage and errors, i.e., MAE value of 0.0098 and RMSE value of 0.0123. The sensitivity analysis results showed that the angles of inclination of the bed of the tilting flume, followed by the number of jets, are the highly influential parameters that affect aeration efficiency.

### Supplementary Information

Supplementary Table S1.

## Data Availability

The data that support the findings in this study are available from the corresponding authors on reasonable request.

## Abbreviations

${C}_{down}$: Dissolved oxygen concentration downstream
${C}_{up}$: Dissolved oxygen concentration upstream
E_20_: Aeration efficiency
J_n_: Number of jets
ANFIS: Adaptive neuro fuzzy inference system
CC: Coefficient of correlation
DO: Dissolved oxygen
df: Degree of freedom
GEP: Genetic expression programming
HR: Cumulative hydraulic radius
HR_Jn_: Hydraulic radius of each jet
*Fr*: Froude number
θ: Angle of inclination
Q: Discharge
ML: Machine learning
R^2^: Coefficient of determination
SVM: Support vector machines
RF: Random forest
ANN: Artificial neural network
MAE: Mean absolute error
RMSE: Root mean square error
