# Supplementary material for: Performance analysis and modelling of circular jets aeration in an open channel using soft computing techniques
Source: Sci Rep. 2024 Feb 7;14:3140. doi: 10.1038/s41598-024-53407-3 (PMC10850504; doi:10.1038/s41598-024-53407-3)
Supplement: Supplementary file 1 — Supplementary Table S1. [file 41598_2024_53407_MOESM1_ESM.docx]

**Supplementary materials**

**for**

**Performance Analysis and Modelling of Circular Jets Aeration in an Open Channel Using Soft Computing Techniques**

**Table S1:** List of the input and output data for the 63 experiments.

| **Sr. No** | **Input** | | | | | | **Output** |
| --- | --- | --- | --- | --- | --- | --- | --- |
|  | θ (°) | Q (L/s) | Jn (Number) | HR (cm) | HR_Jn_(cm) | *Fr* | E_20_ |
| 1 | 0 | 3.41 | 1 | 1.563977 | 1.563977 | 1.415564 | 0.070008 |
| 2 | 0 | 3.41 | 2 | 2.211797 | 1.105898 | 1.683399 | 0.08954 |
| 3 | 0 | 3.41 | 4 | 3.127953 | 0.781988 | 2.00191 | 0.10948 |
| 4 | 0 | 3.41 | 8 | 4.423594 | 0.552949 | 2.380686 | 0.132983 |
| 5 | 0 | 3.41 | 16 | 6.255906 | 0.390994 | 2.831129 | 0.164869 |
| 6 | 0 | 3.41 | 32 | 8.847188 | 0.276475 | 3.366799 | 0.18671 |
| 7 | 0 | 3.41 | 64 | 12.51181 | 0.195497 | 4.003821 | 0.210412 |
| 8 | 0 | 3.84 | 1 | 1.563977 | 1.563977 | 1.594067 | 0.080395 |
| 9 | 0 | 3.84 | 2 | 2.211797 | 1.105898 | 1.895675 | 0.099074 |
| 10 | 0 | 3.84 | 4 | 3.127953 | 0.781988 | 2.254351 | 0.137795 |
| 11 | 0 | 3.84 | 8 | 4.423594 | 0.552949 | 2.68089 | 0.153817 |
| 12 | 0 | 3.84 | 16 | 6.255906 | 0.390994 | 3.188133 | 0.186533 |
| 13 | 0 | 3.84 | 32 | 8.847188 | 0.276475 | 3.791351 | 0.209172 |
| 14 | 0 | 3.84 | 64 | 12.51181 | 0.195497 | 4.508701 | 0.231382 |
| 15 | 0 | 4.75 | 1 | 1.563977 | 1.563977 | 1.971827 | 0.093392 |
| 16 | 0 | 4.75 | 2 | 2.211797 | 1.105898 | 2.344911 | 0.112228 |
| 17 | 0 | 4.75 | 4 | 3.127953 | 0.781988 | 2.788585 | 0.149769 |
| 18 | 0 | 4.75 | 8 | 4.423594 | 0.552949 | 3.316205 | 0.185021 |
| 19 | 0 | 4.75 | 16 | 6.255906 | 0.390994 | 3.943655 | 0.188542 |
| 20 | 0 | 4.75 | 32 | 8.847188 | 0.276475 | 4.689822 | 0.215775 |
| 21 | 0 | 4.75 | 64 | 12.51181 | 0.195497 | 5.57717 | 0.253634 |
| 22 | 1.5 | 3.41 | 1 | 1.563977 | 1.563977 | 1.415564 | 0.079897 |
| 23 | 1.5 | 3.41 | 2 | 2.211797 | 1.105898 | 1.683399 | 0.093681 |
| 24 | 1.5 | 3.41 | 4 | 3.127953 | 0.781988 | 2.00191 | 0.110214 |
| 25 | 1.5 | 3.41 | 8 | 4.423594 | 0.552949 | 2.380686 | 0.159488 |
| 26 | 1.5 | 3.41 | 16 | 6.255906 | 0.390994 | 2.831129 | 0.198082 |
| 27 | 1.5 | 3.41 | 32 | 8.847188 | 0.276475 | 3.366799 | 0.220272 |
| 28 | 1.5 | 3.41 | 64 | 12.51181 | 0.195497 | 4.003821 | 0.232809 |
| 29 | 1.5 | 3.84 | 1 | 1.563977 | 1.563977 | 1.594067 | 0.087694 |
| 30 | 1.5 | 3.84 | 2 | 2.211797 | 1.105898 | 1.895675 | 0.104287 |
| 31 | 1.5 | 3.84 | 4 | 3.127953 | 0.781988 | 2.254351 | 0.149776 |
| 32 | 1.5 | 3.84 | 8 | 4.423594 | 0.552949 | 2.68089 | 0.187001 |
| 33 | 1.5 | 3.84 | 16 | 6.255906 | 0.390994 | 3.188133 | 0.220427 |
| 34 | 1.5 | 3.84 | 32 | 8.847188 | 0.276475 | 3.791351 | 0.231611 |
| 35 | 1.5 | 3.84 | 64 | 12.51181 | 0.195497 | 4.508701 | 0.267521 |
| 36 | 1.5 | 4.75 | 1 | 1.563977 | 1.563977 | 1.971827 | 0.099142 |
| 37 | 1.5 | 4.75 | 2 | 2.211797 | 1.105898 | 2.344911 | 0.120716 |
| 38 | 1.5 | 4.75 | 4 | 3.127953 | 0.781988 | 2.788585 | 0.165753 |
| 39 | 1.5 | 4.75 | 8 | 4.423594 | 0.552949 | 3.316205 | 0.220272 |
| 40 | 1.5 | 4.75 | 16 | 6.255906 | 0.390994 | 3.943655 | 0.233623 |
| 41 | 1.5 | 4.75 | 32 | 8.847188 | 0.276475 | 4.689822 | 0.242813 |
| 42 | 1.5 | 4.75 | 64 | 12.51181 | 0.195497 | 5.57717 | 0.298265 |
| 43 | 3 | 3.41 | 1 | 1.563977 | 1.563977 | 1.415564 | 0.105115 |
| 44 | 3 | 3.41 | 2 | 2.211797 | 1.105898 | 1.683399 | 0.149769 |
| 45 | 3 | 3.41 | 4 | 3.127953 | 0.781988 | 2.00191 | 0.164756 |
| 46 | 3 | 3.41 | 8 | 4.423594 | 0.552949 | 2.380686 | 0.220427 |
| 47 | 3 | 3.41 | 16 | 6.255906 | 0.390994 | 2.831129 | 0.222356 |
| 48 | 3 | 3.41 | 32 | 8.847188 | 0.276475 | 3.366799 | 0.244048 |
| 49 | 3 | 3.41 | 64 | 12.51181 | 0.195497 | 4.003821 | 0.265271 |
| 50 | 3 | 3.84 | 1 | 1.563977 | 1.563977 | 1.594067 | 0.16625 |
| 51 | 3 | 3.84 | 2 | 2.211797 | 1.105898 | 1.895675 | 0.181251 |
| 52 | 3 | 3.84 | 4 | 3.127953 | 0.781988 | 2.254351 | 0.199868 |
| 53 | 3 | 3.84 | 8 | 4.423594 | 0.552949 | 2.68089 | 0.22161 |
| 54 | 3 | 3.84 | 16 | 6.255906 | 0.390994 | 3.188133 | 0.231611 |
| 55 | 3 | 3.84 | 32 | 8.847188 | 0.276475 | 3.791351 | 0.256205 |
| 56 | 3 | 3.84 | 64 | 12.51181 | 0.195497 | 4.508701 | 0.290204 |
| 57 | 3 | 4.75 | 1 | 1.563977 | 1.563977 | 1.971827 | 0.185021 |
| 58 | 3 | 4.75 | 2 | 2.211797 | 1.105898 | 2.344911 | 0.210412 |
| 59 | 3 | 4.75 | 4 | 3.127953 | 0.781988 | 2.788585 | 0.22161 |
| 60 | 3 | 4.75 | 8 | 4.423594 | 0.552949 | 3.316205 | 0.231611 |
| 61 | 3 | 4.75 | 16 | 6.255906 | 0.390994 | 3.943655 | 0.255288 |
| 62 | 3 | 4.75 | 32 | 8.847188 | 0.276475 | 4.689822 | 0.278854 |
| 63 | 3 | 4.75 | 64 | 12.51181 | 0.195497 | 5.57717 | 0.321745 |
